# Midregional-proAtrial Natriuretic Peptide and High Sensitive Troponin T Strongly Predict Adverse Outcome in Patients Undergoing Percutaneous Repair of Mitral Valve Regurgitation

**DOI:** 10.1371/journal.pone.0137464

**Published:** 2015-09-14

**Authors:** Jochen Wöhrle, Mahir Karakas, Ulrike Trepte, Julia Seeger, Birgid Gonska, Wolfgang Koenig, Wolfgang Rottbauer

**Affiliations:** Department of Internal Medicine II–Cardiology, University of Ulm Medical Center, Ulm, Germany; Medical University Hamburg, University Heart Center, GERMANY

## Abstract

**Background:**

It is not known whether biomarkers of hemodynamic stress, myocardial necrosis, and renal function might predict adverse outcome in patients undergoing percutaneous repair of severe mitral valve insufficiency. Thus, we aimed to assess the predictive value of various established and emerging biomarkers for major adverse cardiovascular events (MACE) in these patients.

**Methods:**

Thirty-four patients with symptomatic severe mitral valve insufficiency with a mean STS-Score for mortality of 12.6% and a mean logistic EuroSCORE of 19.7% undergoing MitraClip therapy were prospectively included in this study. Plasma concentrations of mid regional-proatrial natriuretic peptide (MR-proANP), Cystatin C, high-sensitive C-reactive protein (hsCRP), high-sensitive troponin T (hsTnT), N-terminal B-type natriuretic peptide (NT-proBNP), galectin-3, and soluble ST-2 (interleukin 1 receptor-like 1) were measured directly before procedure. MACE was defined as cardiovascular death and hospitalization for heart failure (HF).

**Results:**

During a median follow-up of 211 days (interquartile range 133 to 333 days), 9 patients (26.5%) experienced MACE (death: 7 patients, rehospitalization for HF: 2 patients). Thirty day MACE-rate was 5.9% (death: 2 patients, no rehospitalization for HF). Baseline concentrations of hsTnT (Median 92.6 vs 25.2 ng/L), NT-proBNP (Median 11251 vs 1974 pg/mL) and MR-proANP (Median 755.6 vs 318.3 pmol/L, all p<0.001) were clearly higher in those experiencing an event vs event-free patients, while other clinical variables including STS-Score and logistic EuroSCORE did not differ significantly. In Kaplan-Meier analyses, NT-proBNP and in particular hsTnT and MR-proANP above the median discriminated between those experiencing an event vs event-free patients. This was further corroborated by C-statistics where areas under the ROC curve for prediction of MACE using the respective median values were 0.960 for MR-proANP, 0.907 for NT-proBNP, and 0.822 for hsTnT.

**Conclusions:**

MR-proANP and hsTnT strongly predict cardiovascular death and rehospitalization for HF in patients undergoing percutaneous repair of mitral valve insufficiency. Both markers might be useful components in new scoring systems to better predict short- and potentially long-term mortality and morbidity after MitraClip procedure.

## Introduction

In Europe, mitral valve regurgitation (MR) is the second most frequent valvular disease requiring surgery [1]. According to current guidelines, percutaneous mitral valve repair with the MitraClip procedure may be considered in symptomatic patients with severe primary or secondary MR despite optimal medical therapy, who fulfil the echocardiographic criteria of eligibility, are judged inoperable or at high risk for surgery by a heart team, and who have a life expectancy of > one year. This is supported by recently published 4-year data from the Endovascular Valve Edge-to-Edge REpair Study (EVEREST) trial, showing that, although MitraClip patients more commonly required surgery to treat residual MR (24.8 vs. 5.5%), there was no difference between patients treated percutaneously or surgically with regard to mortality (17.4 vs. 17.8%) and functional improvement (moderate/severe MR: 21.4 vs. 24.7%) [2].

The selection of patients in whom the expected benefits outweigh the risk of the intervention remains challenging. Stratification for adverse cardiovascular outcome among these high-risk individuals is of considerable interest because of the potential to use alternative therapies such as surgical valve repair and valve replacement, or intensified medical therapy. Such efforts have been undertaken by introducing various scores based on clinical and biochemical variables-like the STS-score or the EuroSCORE [3, 4]. It is not known whether biomarkers of hemodynamic stress (e.g. MR-proANP or NT-proBNP), myocardial necrosis (e.g. hsTnT), inflammation and fibrosis (e.g. hsCRP and galectin-3), cardiac biomechanical stress, notably soluble (s)ST2, and renal function (e.g. cystatin C) might improve prediction of adverse outcome in patients undergoing MitraClip procedure.

Thus, we thought to assess the predictive value of various established and emerging biomarkers for major adverse cardiovascular events (MACE) in these patients.

## Materials and Methods

### Study population

This prospective study comprised 34 patients with symptomatic severe mitral regurgitation in whom surgical procedure was considered to be associated with a high risk of mortality, as assessed by STS-Score and logistic EuroSCORE. All patients underwent MitraClip procedure between January 2012 and June 2013.

### Data collection

After admission all subjects filled out a standardized questionnaire containing a medical history and socio-demographic information. In all patients, active follow-up was conducted at day 30 and after a median of 211 days. Information was obtained from patients by personal contact or telephone interview. MACE was defined as cardiovascular death or hospitalization for heart failure (HF). Information regarding adverse cardiovascular disease (CVD) events and treatment since discharge was obtained from the primary care physicians also by means of a standardized questionnaire. If a subject had died during follow-up, the death certificate was obtained from the local Public Health Department and the main cause of death was coded according to the International Classification of Diseases (ICD-9 pos. 390–459: ICD-10 pos. I0-I99 and R57.0). All non-fatal adverse events were reported by the primary care physicians.

Patients were followed according to a protocol approved by the local Ethics Committee of the University of Ulm. All subjects gave written informed consent before baseline investigations were performed and the study was conducted in accordance with the Helsinki declaration.

### Laboratory methods

Blood was drawn at baseline immediately before the MitraClip procedure in a fasting state under standardized conditions and stored at -80°C until analysis. MR-proANP concentrations were measured with an automated sandwich chemiluminescence immunoassay on a KRYPTOR System (BRAHMS AG, Hennigsdorf/Berlin, Germany) [5]. The inter-assay coefficients of variation (CV) were 7.0% and 3.8% at 92.3 and 486 pmol/L. NT-proBNP as well as hsTnT was measured by electrochemiluminescence on a Cobas e411 [6] (Roche Diagnostics, Penzberg, Germany). The inter-assay CV was 5.1% and 2.2% at 135 and 5364 pg/mL. For hsTnT a value of 14 ng/L has recently been reported to represent the 99^th^ percentile in a healthy reference population [7]. The inter-assay CVs were 4.3% and 3.4% at concentrations of 29 and 2309 ng/L. C-reactive protein (CRP) concentrations were measured by latex enhanced immunonephelometry on a Behring Nephelometer II (N Latex CRP mono Dade-Behring, Marburg) [8]. Inter-assay CV was 4.1%. Cystatin C was determined on the same device [9] (Dade-Behring, Marburg). Inter-assay CV was 4.0%. SST-2 levels were determined using a second‐generation, high‐sensitivity enzyme‐linked immunosorbent assay (ELISA) (Critical Diagnostics, detection limit 2 ng/mL), while galectin-3 levels were determined using an ELISA from BG Medicine. Inter-assay CVs were 7.6% for sST-2 and 3.5% for Galectin-3, respectively. All biomarkers were measured in a blinded fashion. Standard laboratory parameters were done by routine methods.

### Statistical methods

The study population has been described with respect to various sociodemographic and medical characteristics. Normally distributed, continuous variables are expressed as arithmethic mean (±SD). Non-normally distributed variables are given as medians [interquartile range]. Differences between mean values were analysed using the Student’s t-test and those between median values by the Mann-Whitney U test. Chi square test was used for categorical parameters. For all analyses a p-value <0.05 was considered to be statistically significant. The relation between biomarker concentrations and future events was assessed by the Kaplan-Meier and life table method and quantified by means of the log-rank test. The Receiver-Operating Characteristic (ROC) analysis was used to assess the area under the curve (AUC) to determine discrimination between events and event-free patients. All statistical evaluations were performed using the MedCalc software package (Version 13.1, 2014).

## Results

Procedural success was achieved in all patients (reduction of MR to grade ≤2+. Table 1 shows the main sociodemographic and laboratory characteristics in all 34 patients undergoing the MitraClip procedure, of whom those experiencing MACE are displayed as events. During a median follow-up of 211 days (interquartile range 133 to 333 days), 9 patients (26.5%) experienced MACE (death: 7 patients, rehospitalization for HF: 2 patients). Thirty days MACE-rate was 5.9% (death: 2 patients, no rehospitalization for HF).

**Table 1:** Baseline characteristics in 34 patients undergoing MitraClip procedure, of whom 9 experienced MACE.

	Events	Non-Events	p-value
Number	9	25	n/a
Age [years]	79.6 (8.6)	79.4 (6.7)	0.95
Hypertension [%]	78	80	0.73
Hyperlipidemia [%]	67	64	0.96
Type 2 Diabetes [%]	33	24	0.92
Current or former smoker [%]	33	20	0.77
Male Sex [%]	56	52	0.84
Known CHD [%]	89	84	0.85
NYHA Classification [%]			0.86
III	33	36	
III-IV	33	24	
IV	33	40	
Degree of insufficiency (1–3) Median	3	3	0.96
Left-ventricular ejection fraction [%]			0.30
Above 50%	33	68	
Between 30%-50%	56	28	
Below 30%	11	4	
Atrial Fibrillation [%]	67	48	0.57
Etiology of Mitral Insufficiency [%]			0.12
Degenerative	56	88	
Functional	44	12	
Logistic Euro Score [%]			0.15
Mean	27.8 (19.4)	16.9 (16.5)	
Median	26.8 [12.3; 41.7]	11.9 [6.8; 21.6]	
STS-Score [%]			0.06
Mean Risk for Mortality	17.1 (11.0)	11.1 (7.3)	
Median Risk for Mortality	14.3 [12.0; 22.6]	9.9 [5.8; 12.7]	
NT-proBNP [pg/mL]			***0*.*0004***
Mean	12,705 (10,702)	2,168 (1,602)	
Median	11,251 [3619; 19137]	1,974 [1051; 2940]	
MR-proANP [pmol/L]			***0*.*0001***
Mean	757.1 (298.2)	330.37 (127.66)	
Median	755.6 [570.2; 800.6]	318.30 [261.4; 387.2]	
Hs-TnT [ng/L]			***0*.*0047***
Mean	267.5 (534.8)	36.55 (33.3)	
Median	92.6 [38.0; 155.5]	25.20 [16.4; 48.7]	
Hs-CRP [mg/L]			***0*.*0334***
Mean	37.6 (66.9)	6.8 (7.6)	
Median	14.6 [4.3; 32.0]	5.5 [1.5; 8.3]	
Cystatin C [mg/L]			***0*.*0112***
Mean	2.2 (0.7)	1.5 (0.6)	
Median	2.1 [1.7; 2.6]	1.4 [1.2; 1.7]	
Galectin-3 [ng/mL]			***0*.*0007***
Mean	37.4 (14.0)	19.2 (9.7)	
Median	34.8 [26.1; 48.1]	16.6 [12.9; 20.6]	
SST-2 [ng/mL]			***0*.*0025***
Mean	119.9 (116.4)	37.3 (20.6)	
Median	75.1 [52.7; 134.8]	29.4 [24.9; 43.6]	

Clinical variables, including NYHA classification, aetiology and degree of mitral valve insufficiency, left-ventricular ejection fraction, STS-Score and logistic EuroSCORE did not differ significantly between groups.

In contrast, baseline biomarker concentrations were clearly higher in those experiencing an event vs event-free patients. Levels of hsTnT (median 92.6 vs 25.2 ng/L), NT-proBNP (median 11251 vs 1974 pg/mL), MR-proANP (median 755.6 vs 318.3 pmol/L, all p<0.005) showed the most pronounced differences between both groups.

Table 2 summarizes the prognostic performance of the various biomarkers. Of all biomarkers measured, NT-proBNP and MR-proANP outperformed the others, although hsTnT, galectin-3 and sST-2 also performed well with AUCs above 0.8. MR-proANP above the ROC-optimized cut-off of 448.4 pmol/L would have correctly predicted MACE, with a sensitivity of 100%, and a specificity of 84%.

By comparison, hsTnT, using the ROC-optimized cut-off of 32.8 ng/L, would have correctly predicted MACE with a lower sensitivity of 88.9% and specificity of 68%.

**Table 2:** Predictive ability of the biomarkers measured as assessed by area under the ROC curve analyses.

Biomarker	Median in 34 patients	ROC-optimized cut-off	Sensitivity [%]	Specificity [%]	Area under the ROC-Curve	AUC_ROC 95% CI	p (Area = 0.5) =
NT-proBNP [pg/mL]	2558	>3739	77.8	92.0	0.907	0.757–0.979	***<0*.*0001***
MR-proANP [pmol/L]	361.4	>448.4	100	84.0	0.960	0.831–0.998	***<0*.*0001***
HsTnT [ng/L]	32.6	>32.8	88.9	68.0	0.822	0.653–0.932	***0*.*0003***
HsCRP [mg/L]	6.43	>14	55.6	92.0	0.742	0.564–0.876	***0*.*0227***
Cystatin C [mg/L]	1.57	>1.67	88.9	76.0	0.789	0.615–0.909	***0*.*0048***
Galectin-3 [ng/mL]	36.4	>22.6	88.9	80.0	0.884	0.728–0.968	***<0*.*0001***
SST-2 [ng/mL]	19.2	>52.8	77.8	88.0	0.884	0.679–0.945	***0*.*0002***

Table 3 shows the results for superiority testing of the MR-proANP ROC curve in comparison with the other biomarkers. Of all biomarkers tested, statistically significant superiority was achieved in comparison to hsCRP, while a comparative analysis yielded borderline significance for superiority of MR-proANP against cystatin C and hsTnT.

**Table 3:** Pairwise comparison of ROC curves (MR-proANP vs other biomarkers).

Comparator	Difference between areas	Standard error	95% CI	p for superiority of MR-proANP =
NT-proBNP	0.053	0.051	-0.0472 to 0.154	0.2985
HsTnT	0.138	0.075	-0.00963 to 0.285	0.0670
HsCRP	0.218	0.105	0.0122 to 0.423	***0*.*0378***
Cystatin C	0.171	0.094	-0.0138 to 0.356	0.0698
Galectin-3	0.076	0.059	-0.0407 to 0.192	0.2026
SST-2	0.116	0.080	-0.0417 to 0.273	0.1499

**Fig 1:**
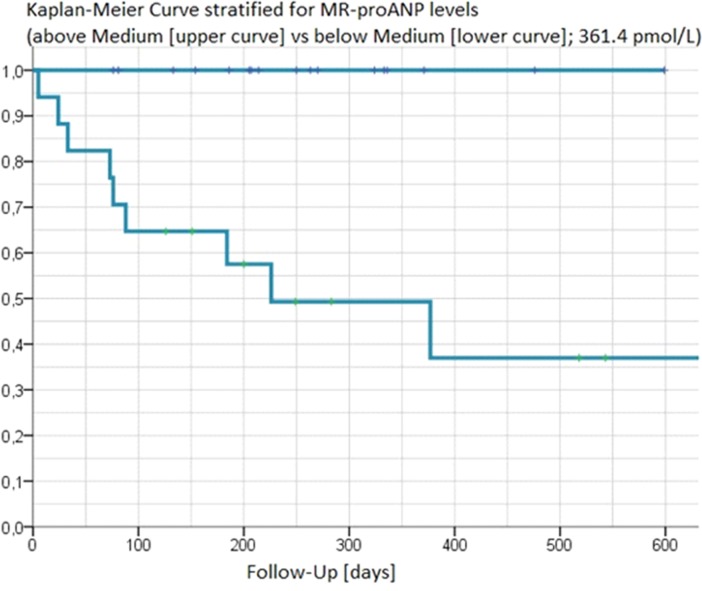
Kaplan-Meier curves of MR-proANP above and below the median [y-axis describing freedom from CV death or hospitalisation for heart failure].

**Fig 2:**
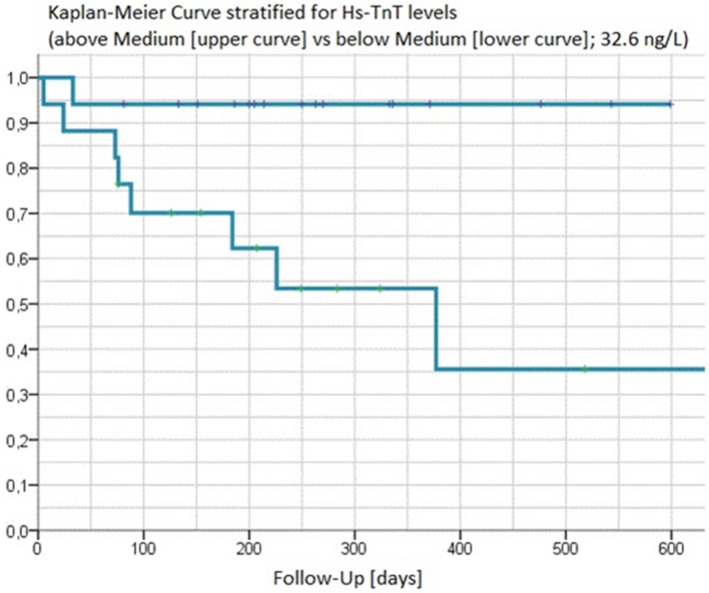
Kaplan-Meier curves of hsTnT above and below the median [y-axis describing freedom from CV death or hospitalisation for heart failure].

In Kaplan-Meier analyses values for MR-proANP (Fig 1) and hsTnT (Fig 2) above the median clearly separated those experiencing an event vs. event-free patients (p-value for log-rank test 0.0015 and 0.0109, respectively). This was further corroborated by C-statistics (Table 2) where areas under the ROC curve for prediction of MACE using the respective median values were 0.960 for MR-proANP, and 0.822 for hsTnT.

## Discussion

To our knowledge, this is the first study that evaluates the predictive role of a comprehensive multi-marker panel in patients undergoing percutaneous repair of mitral valve insufficiency. Two other MitraClip studies recently proposed clinical algorithms for patient selection, and indicated that impairment of left-ventricular ejection fraction and massively elevated NT-proBNP values predict mortality in patients with severe MR [10, 11].

In our prospective study, elevated MR-proANP was the strongest predictor of outcome, and risk of adverse cardiovascular events after MitraClip procedure was clearly higher in patients with MR-proANP concentrations above the median. Impressively, while clinical characteristics were not statistically significantly different between patients with events and patients without events, a single MR-proANP measurement at the time of MitraClip implantation identified all MACE as documented by Kaplan-Meier analyses (p-value for log-rank test 0.0015).

Since only three out of thirty-four patients in our study suffered from functional mitral insufficiency, our results should only be applied to mitral insufficiency of degenerative etiology.

### MR-proANP in comparison with NT-proBNP

MR-proANP is equivalent to BNP or NT-proBNP in the diagnosis of acute HF in patients presenting with shortness of breath to the emergency department and has similar biological effects as BNP [12]. In current European guidelines both peptides are considered equal for diagnosis of chronic and acute HF [13]. In the BACH trial, a prospective study of 1,641 patients presenting to the emergency department with dyspnea, MR-proANP (levels above 120 pmol/l) proved non-inferior to NT-proBNP (levels above 100 pg/ml) for the diagnosis of acute HF [10]. In our study AUC for a MR-proANP based risk prediction was higher than a NT-proBNP based model (AUC 0.960 versus 0.907). Three issues may be considered here:

First, the synthesis, secretion and clearance of BNP differ from those of ANP, suggesting discrete physiological and pathophysiological roles of both peptides [14, 15]. ANP secretion occurs from a previously synthesized pool in the secretory granules of atrial cardiomyocytes. Therefore, the regulation of ANP release occurs mainly at the level of hormone secretion, while in contrast most BNP regulation is done during gene expression, with the majority of BNP being synthesized during bursts of activation from pathophysiological stimuli [16].

Second, the natriuretic response may vary depending on the type of pathophysiological stress. For example, in the early phase of ACS, and in human cardiac allograft acute rejection, BNP gene expression increases considerably, whereas ANP levels increase only slightly [17]. In contrast, available data suggest, that ANP is much more sensitive in subclinical disease [18]. For example, in cardiac hypertrophy, plasma ANP levels increase as atrial pressure increases, whereas BNP plasma levels rise when ventricular hypertrophy develops [19].

Third, ischemic conditions, as evidenced by a median hsTnT of 32.6 ng/L in our cohort, directly stimulate the natriuretic system-but again, ANP seems to be the more sensitive peptide. Accumulated experimental data clearly show that hypoxia leads to an increased synthesis of ANP in both the normal, and the hypertrophied myocardium [20]. In cultured atrial myocytes without any stretch changes, hypoxia stimulated ANP gene expression, and a region of hypoxia-response elements could be characterized within the ANP gene promoter. Although hypoxia-response elements have also been found in the promoter sequence of the BNP gene, and it has been shown that hypoxia also stimulates the release of BNP, data from perfused rat ventricular myocardium showed that hypoxia-stimulated ANP release was more pronounced than release of BNP [21].

### High sensitive troponin

After establishment as diagnostic biomarkers in the setting of an acute coronary syndrome (ACS) most recently approved high-sensitive assays are able to predict long-term cardiovascular prognosis even in healthy subjects and stable coronary heart disease (CHD) patients [22]. Elevated hsTnT, above the median, as an established marker of myocardial injury, showed similar results in Kaplan-Meier analyses as MR-proANP and identified 8 out of 9 MACE, although being inferior to MR-proANP in C-statistics.

### Biomarkers of renal dysfunction, inflammation, fibrosis, and cardiac biomechanical stress

During recent years evidence has been accumulated for cystatin C, which emerged as a novel renal biomarker with prognostic implications in patients with acute and chronic HF [23]. Although showing a good sensitivity (88.9%) for prediction of MACE, the AUC using ROC-optimized cut-points was at the lower end of markers tested (0.789), which seems plausible, since cystatin C has no capacity in reflecting hemodynamic stress.

HsCRP, a widely established biomarker to improve risk prediction for CVD showed the lowest performance (AUC 0.742) in this multi-marker panel, and was the only biomarker to be statistically significantly inferior to MR-proANP in pairwise comparison of ROC curves [8].

Soluble ST-2 is an anti‐hypertrophic protein, and a member of the interleukin-1 receptor family, which also reflects myocyte stretch in experimental studies [24]. It has emerged as a strong prognostic biomarker in patients with HF and myocardial infarction, and just recently, increased sST2 in patients with stable CHD in the LURIC study has been shown to be an independent predictor of long-term all-cause mortality and to provide complementary prognostic information to hsTnT and NT-proBNP [25].

Galectin-3 is a biomarker representing an integrated pathophysiologic signal for myocardial fibrosis and ventricular dysfunction. Higher levels of circulating galectin-3 have been associated with increased risk for incident HF, adverse left ventricular remodelling, decompensated HF, and worse prognosis [26]. In accordance with the accumulated evidence regarding their predictive ability in CVD, sST2 and galectin-3 predicted well in our MitraClip cohort (AUC 0.884 for both markers), although lower than MR-proANP. This seems plausible: mitral regurgitation burdens the left ventricle with inflammation (mirrored by hsCRP and sST2) and fibrosis (mirrored by Galectin-3) in the early clinical course, but these mechanisms are gradually replaced by chronic remodelling processes with enlargement of the LV chamber, which are more appropriately represented by natriuretic peptides. HsCRP, sST2, and galectin-3 have only limited capacity in mirroring hemodynamic stress.

## Conclusions

Our prospective study shows that MR-proANP and hsTnT strongly predict cardiovascular death and rehospitalization for HF in patients undergoing percutaneous repair of mitral valve insufficiency. Both markers might represent useful components in new scoring systems to better predict short- and potentially long-term mortality and morbidity after MitraClip procedure. Larger and better controlled independent studies are needed to confirm these results.
